# Experimental Susceptibility of *Nyssomyia antunesi* and *Lutzomyia longipalpis* (Psychodidae: Phlebotominae) to *Leishmania* (*Viannia*) *lainsoni* and *L.* (*V.*) *lindenbergi* (Trypanosomatidae: Leishmaniinae)

**DOI:** 10.3390/microorganisms12040809

**Published:** 2024-04-17

**Authors:** Yetsenia del Valle Sánchez Uzcátegui, Fernando Tobias Silveira, Thais Gouvea de Morais, Rodrigo Ribeiro Furtado, Thiago Vasconcelos dos Santos, Marinete Marins Póvoa

**Affiliations:** 1 Programa de Pós Graduação em Biologia de Agentes Infecciosos e Parasitários, Instituto de Ciências Biológicas, Universidade Federal do Pará, Belém 66075-110, PA, Brazil; 2 Seção de Parasitologia, Instituto Evandro Chagas, Ananindeua 67030-000, PA, Brazil; 3 Departamento de Biología, Facultad de Ciencias, Universidad de Los Andes, Mérida 5101, Venezuela

**Keywords:** *Nyssomyia antunesi*, *Lutzomyia longipalpis*, *Leishmania lainsoni*, *Leishmania lindenbergi*, experimental infection

## Abstract

The present work assessed the experimental susceptibility of *Nyssomyia antunesi* and *Lutzomyia longipalpis* to *Leishmania* (*Viannia*) *lainsoni* and *L.* (*V*.) *lindenbergi*. A *L.* (*Leishmania*) *chagasi*–*Lu. longipalpis* combination was used as a susceptible control. Wild-caught *Ny. antunesi* and laboratory-bred *Lu. longipalpis* were membrane-fed on blood with a 5 × 10^6^/mL log-phase promastigote culture suspension and dissected on days 2 and 8 post-blood meal (pbm) for analysis focused on the assessment of parasitoses, as well as placement and promastigote morphotyping. Survival curves were constructed. In all combinations, promastigotes were observed on day 8 pbm. For both *Leishmania* species, in *Lu. longipalpis*, the presence of parasites was observed up to the stomodeal valve, while in *Ny. antunesi*, the presence of parasites was observed up to the cardia. There were no significant differences in parasitosis between *L.* (*V.*) *lainsoni* and *L.* (*V.*) *lindenbergi* in either *Ny. antunesi* or *Lu. longipalpis*. Six morphological promastigote forms were distinguished in Giemsa-stained gut smears. The survival curves of all combinations decreased and were affected differently by several *Lu. longipalpis*–parasite combinations, as well with *Lu. longipalpis*–uninfected blood. These findings stress *Lu. longipalpis* as experimentally susceptible to *Leishmania* spp. and suggest the putative susceptibility of *Ny. antunesi* to *L.* (*V.*) *lainsoni* and *L.* (*V.*) *lindenbergi*.

## 1. Introduction

Phlebotomines (Diptera: Psychodidae) are medically important insects implicated in the transmission of several pathogens, mainly *Leishmania* protozoa (Kinetoplastea: Trypanosomatidae) [1], which are the agents of leishmaniases, a group of neglected tropical diseases affecting millions of people worldwide [2].

*Leishmania* parasites have a digenetic life cycle and infect a wide range of vertebrate reservoir hosts and invertebrate vectors, mainly phlebotomines (Diptera: Psychodidae) [3,4,5]. The development of *Leishmania* within the phlebotomines is a complex process: after the ingestion of infected blood, amastigotes (nonflagellated forms) differ from promastigotes (flagellated forms) inside the insect’s gut [6], overcoming adverse conditions such as physico-chemical barriers and excretion flow [7,8,9,10]. Several subtypes of promastigote forms are recognized according to their morphology, including procyclic, haptomone, nectomonad, paramastigote and metacyclic [11], the latter of which are infective to vertebrate hosts [12,13].

A fundamental aspect in determining whether phlebotomines are incriminated by the transmission of *Leishmania* is the differentiation of infectious metacyclic forms [14]. In this same sense, the parasitosis and placement of late-stage infection constitute important parameters for evaluating the vector competence of a particular phlebotomine species for the developmental success of a given *Leishmania* sp. [15].

In the Brazilian Amazon, a particular tegumentary leishmaniasis (TL) transmission scenario occurs mainly because of the etiology of *L.* (*Leishmania*) *amazonensis*, *L.* (*Viannia*) *lainsoni* and *L.* (*V*.) *lindenbergi* in the forest fragments of Belém city [16]. In these TL *foci*, with respect to *L.* (*L*.) *amazonensis, Bichromomyia flaviscutellata* has well-established vector evidence [17]; for *L.* (*V*.) *lainsoni,* species of *Trichophoromyia*, particularly *Th. Ubiquitalis* and *Th. brachipyga*, have been shown to be involved in transmission [18]; ultimately, for *L.* (*V*.) *lindenbergi, Nyssomyia antunesi* has received attention due to its abundance, dominance, spatiotemporal convergence with human disease, blood feeding on human and potential reservoirs of *Leishmania* [19,20]. Despite the lack of evidence of true and species-specific identifiable *Leishmania* infection, the vector role of *Ny. antunesi* remains undefined, not advancing on suspect status.

However, studies on the interactions between *Leishmania* and its vectors are required to advance the understanding of the processes involved in parasite development and transmission [6]. Some parasite–vector combinations have been studied under laboratory conditions; however, the majority of binomials inferred by field evidence still require laboratory investigation. Therefore, the present study aimed to fill the gap in vector knowledge on the development of *L.* (*V*.) *lainsoni* and *L.* (*V*.) *lindenbergi* in *Ny. antunesi* and *Lu. longipalpis*. A *L.* (*L*.) *chagasi*–*Lu. longipalpis* combination was used as a ‘positive control’.

## 2. Materials and Methods

### 2.1. Parasites

The World Health Organization reference strains of three different *Leishmania* species maintained in the cryobank of the ‘Ralph Lainson’ Leishmaniases Laboratory, Instituto Evandro Chagas (IEC), Belém, Brazil, were used: *L.* (*V.*) *lainsoni* (MHOM/BR/1981/M6426), *L.* (*V.*) *lindenbergi* (MHOM/BR/1996/M15729) and *L.* (*L.*) *chagasi* (MHOM/BR/1981/M6445). Promastigotes were cultured in Schneider’s insect medium (SIM) supplemented with 100 U/mL penicillin, 100 g/mL streptomycin and 10% heat-inactivated fetal bovine serum. To carry out the subsequent experimental infection of phlebotomines, low-passage parasites were used. Before being mixed with the blood, the samples were washed by centrifugation (2400× *g* for 5 min) and resuspended in a sterile container with a saline solution [5].

### 2.2. Phlebotomines

Wild-caught *Ny. antunesi* were obtained from the Bosque Rodrigues Alves-Jardim Botânico da Amazônia (1°25′48″ S; 48°27′25″ W), an urban park of Belém city in which the phlebotomine fauna has already been surveyed [21]. Captures were performed with CDC light traps set 1.5 m above ground level (*n* = 4) and 20 m above ground level (*n* = 2), operating from 6:00 p.m. to 6:00 a.m., from May to August 2023. The phlebotomines were visually screened, aspirated from the primary cage in the field, and transported to the laboratory under 80 ± 10% relative humidity and 10% glucose solution offered ad libitum [22,23]. The phlebotomines were immediately transferred to a secondary nylon cage. Congested, gravid or semigravid females were excluded from the experiments [24].

Laboratory-bred *Lu. longipalpis* from an established Amazonian closed colony (Abaetetuba F236) were used. For the tests, adult female specimens 5–9 days old were used [5,25] and supplied with 10% glucose solution ad libitum [26] up to 24 h before the assays [27,28].

### 2.3. Parasite–Vector Systems

Experimental infections were carried out according to the artificial blood feeding protocol proposed by Sánchez Uzcátegui et al. [24]. Briefly, the groups of both wild-caught *Ny. antunesi* and laboratory-bred *Lu. longipalpis* were artificially fed in 30 cm^3^ nylon cages for 3 h through a sausage membrane installed in a circulator device containing previously heat-inactivated serum (56 °C for 1 h) human blood and 5 × 10^6^/mL promastigotes [5] from log-phase cultures [29]. For *Lu. longipalpis*, females that had fed on uninfected blood were also assessed. The engorged females were confined to 200 mL flasks, and the recipients were lined with moistened filter paper and given a 10% sucrose diet until dissection [5]. Females from each species/experiment were divided into two groups for dissection: one group was dissected before the females defecated (early stage of infection) on day 2 post-blood meal (pbm), and the other group was dissected after defecation (late stage of infection) on day 8 pbm [5,25].

### 2.4. Parasite Detection and Development

Phlebotomines were monitored daily to account for dead females, and survival curves were constructed. Dead females were dissected, and only *Leishmania*-positive females were counted [30]. The proportion survived (lx) was calculated according to Rabinovich [31]. Survivorship curves were obtained for different parasite–vector combinations and were compared by the log-rank test using BioEstat 5.3 software [32]. On days 2 and 8 pbm, the females were removed from the oviposition glasses using a Castro aspirator, and placed at 4 °C for thermal immobilization by cooling. The females were washed once with a 0.9% NaCl solution plus 5% neutral detergent, and twice with 0.9% NaCl for subsequent dissection. Phlebotomines were placed in a drop of phosphate-buffered saline (PBS) on a microscope slide, and the head was separated from the thorax before the intestine was extracted through the apex of the abdomen. The intestines were individually observed under an optical microscope and examined to determine the development of flagellates in the guts [33] following the taxonomic statement of Lainson and Shaw [34], and classified with the semiquantitative parasitosis scale described by Myskova et al. [15], whereby parasite loads were graded as absent (0 parasites per gut), weak (less than 100 parasites per gut), moderate (100–1000 parasites per gut) or heavy (more than 1000 parasites per gut). Promastigotes from the gut were Giemsa-stained and microscopically assessed to infer distinguishable evolutive forms based on morphologic/morphometric criteria modified by Ticha et al. [5], focusing on identifying metacyclic-like forms on day 8 pbm. All experiments were repeated at least three times for each parasite–vector combination. Parasitoses were compared using the G test with BioEstat 5.3 software [32]. In all statistical analysis, *p* ≤ 0.05 was considered to indicate a 95% confidence interval.

### 2.5. Ethical Approval

The capture and processing of invertebrate fauna (phlebotomines) were authorized by the ‘Sistema de Autorização e Informação em Biodiversidade’ under protocol no. 70142-2. Animals used for the blood feeding of phlebotomine colonies were maintained and handled at the Instituto Evandro Chagas animal facility, in accordance with institutional guidelines and Brazilian legislation (Federal Law no. 11.794, 8 October 2008). In vivo blood feeding standard operational procedures were approved by the Ethics Committee on Animal Use (CEUA/IEC), under certificate no. 30/2021.

## 3. Results

### 3.1. Susceptibility of Ny. antunesi to L. (V.) lainsoni and L. (V.) lindenbergi

**Figure 1:**
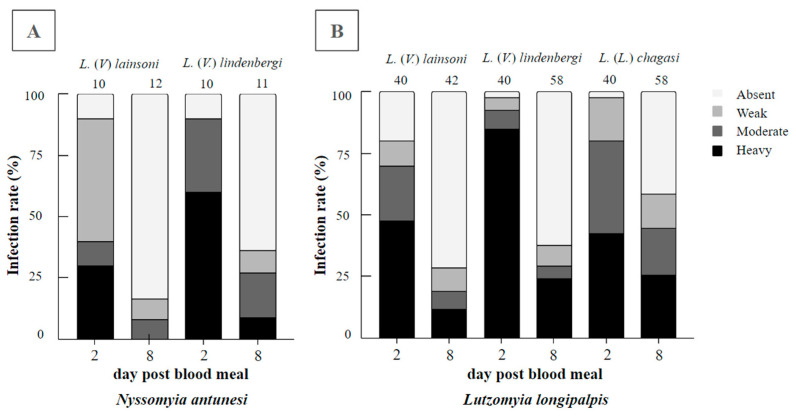
Infection rates and parasitoses of *Leishmania* (*Viannia*) *lindenbergi* (MHOM/BR/1996/M15729) and *L.* (*V.*) *lainsoni* (MHOM/BR/1981/M6426) in (**A**) *Nyssomyia antunesi* and (**B**) *Lutzomyia longipalpis*. The well-known susceptible *L.* (*L.*) *chagasi–Lu. longipalpis* combination was also performed as the control. Intestines were dissected on days 2 and 8 post-blood meal (pbm). Parasitoses were classified into four categories: weak (less than 100 parasites per gut), moderate (100–1000 parasites per gut) and heavy (more than 1000 parasites per gut). The number of females evaluated can be found above the columns.

In total, 43 blood-fed *Ny. antunesi* females were dissected, 22 of which were exposed to *L.* (*V.*) *lainsoni* and 21 to *L.* (*V.*) *lindenbergi*. On day 2 pbm, the infection rates were 90% for both parasite–vector combinations, recording weak, moderate and heavy parasitoses, respectively; 50%, 10% and 30%, respectively, for *L.* (*V*.) *lainsoni*; and 0%, 30% and 60%, respectively, for *L.* (*V.*) *lindenbergi*. Promastigotes were limited to the endoperitrophic space within the ingested blood meal. On day 8 pbm, the infection rates were 16% and 36% for *L.* (*V.*) *lainsoni* and *L.* (*V.*) *lindenbergi*, respectively. For *L.* (*V.*) *lainsoni*, parasitoses were assessed as 8% weak and 8% moderate, whereas for *L.* (*V.*) *lindenbergi*, parasitoses were assessed as 9% weak, 18% moderate and 9% heavy (Figure 1A). The results from the assessment of parasitoses between these *Leishmania* species were not significant (G test = 2.2148, df = 3, *p* = 0.5290).

**Figure 2:**
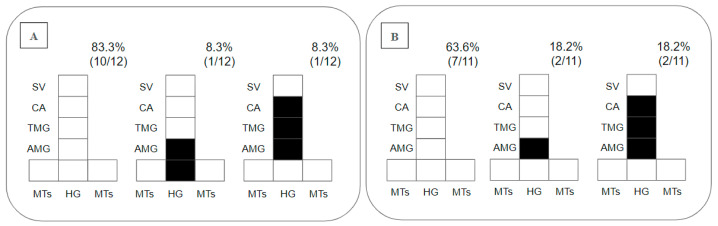
Diagramatic placement of *Leishmania* spp. in the gut of *Nyssomyia antunesi* on day 8 post-blood meal (pbm). (**A**): *Leishmania* (*Viannia*) *lainsoni* (MHOM/BR/1981/M6426); (**B**): *L.* (*V.*) *lindenbergi* (MHOM/BR/1996/M15729). HG, hindgut; MTs, Malpighian tubules; AMG, abdominal midgut; TMG, thoracic midgut; CA, cardia; SV, stomodeal valve. Percent distribution of localization patterns among the infected females is shown in the top right of each diagram.

Regarding the placement of parasites in the gut, as promastigotes were limited to the endoperitrophic space on day 2 pbm, this parameter was only considered on day 8 pbm, when 83% of the *Ny. antunesi–L.* (*V.*) *lainsoni* combinations did not sustain infection; 8.3% presented peripylarian development with colonization in the hindgut (HG) and abdominal midgut (AMG); and 8.3% presented suprapylarian development, with colonization in the AMG, thoracic midgut (TMG) and cardia (CA) (Figure 2A). In *Ny. antunesi–L.* (*V.*) *lindenbergi* combinations, 63.6% of the strains did not sustain infection, and 36% presented suprapylarian development, 18.2% colonization in the AMG and 18.2% suprapylarian development with colonization in the AMG, TMG and CA (Figure 2B).

### 3.2. Susceptibility of Lu. longipalpis to L. (V.) lainsoni and L. (V.) lindenbergi

In total, 180 blood-fed *Lu. longipalpis* females were dissected, 82 were exposed to *L.* (*V.*) *lainsoni* and 98 were exposed to *L.* (*V.*) *lindenbergi*. On day 2 pbm, the infection rates were 81% for *L.* (*V.*) *lainsoni* and 98% for *L.* (*V.*) *lindenbergi*, with promastigotes found in the endoperitrophic space only within the ingested blood meal. Parasitoses were assessed as 10% weak, 23% moderate and 48% heavy for *L. (V.) lainsoni*, whereas parasitosis were assessed as 5% weak, 8% moderate and 85% heavy for *L.* (*V.*) *lindenbergi*. On day 8 pbm, the infection rates were 29% for *L.* (*V.*) *lainsoni* and 38% for *L.* (*V.*) *lindenbergi*, with parasitoses assessed as 10% weak, 7% moderate and 12% heavy for *L.* (*V*.) *lainsoni*, whereas parasitosis were assessed as 9% weak, 5% moderate and 24% heavy for *L.* (*V.*) *lindenbergi* (Figure 1B). The results from the assessment of parasitoses between these *Leishmania* species were not significant (G test = 1.7129, df = 3, *p* = 0.6341).

**Figure 3:**
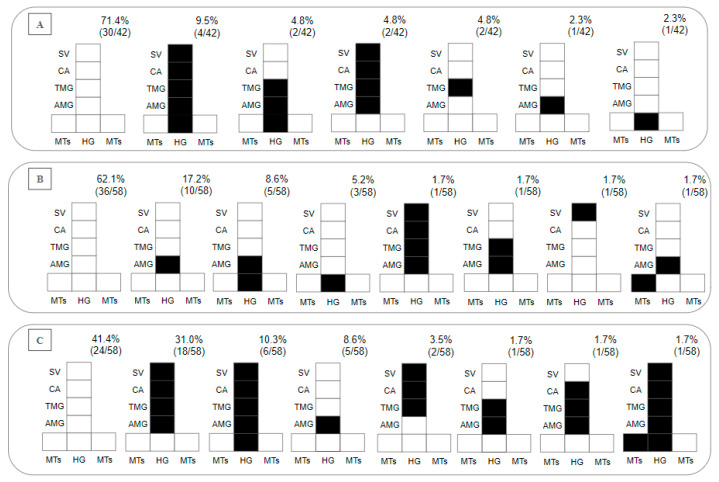
Diagramatic placement of (**A**) *Leishmania* (*Viannia*) *lainsoni* (MHOM/BR/1981/M6426), (**B**) *L.* (*V.*) *lindenbergi* (MHOM/BR/1996/M15729) and (**C**) *L.* (*L*.) *chagasi* (MHOM/BR/1981/M6445) in the gut of *Lu. longipalpis* on the 8th day post-blood meal (pbm). HG, hindgut; MTs, Malpighian tubules; AMG, abdominal midgut; TMG, thoracic midgut; CA, cardia; SV, stomodeal valve. In the upper right part of each diagram is the percentage distribution obtained for each *Leishmania* species within the intestine of *Lu. longipalpis*.

Regarding the presence of parasites in the gut on day 8 pbm, within 28.7% of the *Lu. longipalpis–L.* (*V.*) *lainsoni* positive combinations, 2.4% presented with hypopylarian, 14.3% with peripylarian and 12% with suprapylarian development. The peripylarian patterns included colonization in the HG, AMG, TMG, CA and stomodeal valve (SV) (9.5%), and in the HG, AMG and TMG (4.8%). The suprapylarian patterns included colonization in the AMG, TMG, CA and SV (4.8%), and in the TMG (4.8%) and AMG (2.3%) (Figure 3A). Within 37.8% of the *Lu. longipalpis–L.* (*V.*) *lindenbergi* positive combinations, hypopylarian (5.2%), peripylarian (10.3%) and suprapylarian (22.3%) development was observed. The hipopylarian pattern included colonization in the HG (5.2%); peripylarian comprised colonization in the HG and AMG (8.6%), and MTs and AMG (1.7%); and more frequent suprapylarian colonization was in the AMG (17.2%), while other infection patterns did not exceed 2% (Figure 3B).

### 3.3. Susceptibility of Lu. longipalpis to L. (L.) chagasi (Control Experiment)

In total, 98 *Lu. longipalpis* exposed to *L.* (*L.*) *chagasi* were dissected. On day 2 pbm, the infection rate was 99%, recording 18% weak, 38% moderate and 43% heavy parasitoses. On day 8 pbm, the infection rate was 59%, recording 14% weak, 19% moderate and 26% heavy parasitoses. Peripylarian (12.1%) and suprapylarian (44.8%) development was observed. Peripylarian pattern comprised colonization in the HG, AMG, TMG, CA and SV (10.3%); suprapylarian comprised colonization in the AMG, TMG, CA and SV (31%), exclusively in the AMG (8.6%), and TMG, CA and SV (3.5%); and other infection patterns did not exceed 2% (Figure 3C).

On the other hand, the results of the evaluation of parasites among the *Leishmania* species were significant on day 8 pbm (Table 1).

### 3.4. Morphology of L. (V.) lainsoni and L. (V.) lindenbergi Promastigotes in Ny. antunesi

**Figure 4:**
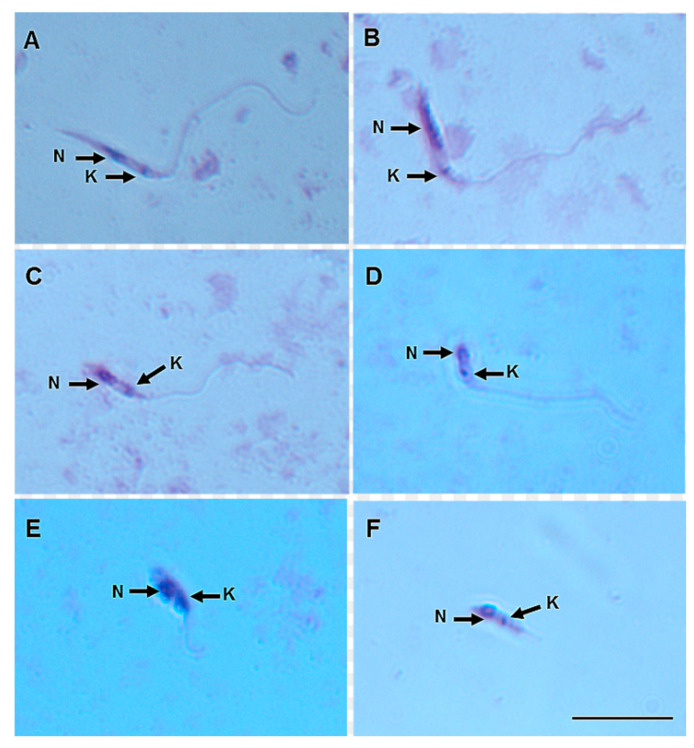
*Leishmania* (*Viannia*) *lindenbergi* (MHOM/BR/96/M15729) morphological form development in the gut of *Nyssomyia antunesi*: (**A**) elongated nectomonad; (**B**) short nectomonad; (**C**) metacyclic promastigote; (**D**) rounded metacyclic promastigote; (**E**) rounded paramastigote; (**F**) haptomonad. N, nucleus; K, kinetoplast (stained by Giemsa). Bar = 10 μm.

**Figure 5:**
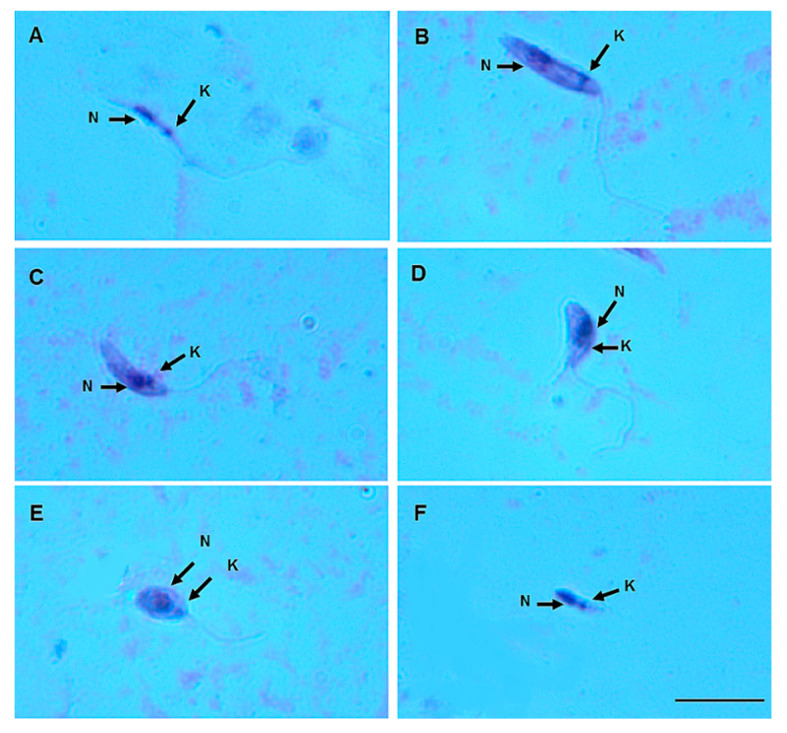
*Leishmania* (*Viannia*) *lainsoni* (MHOM/BR/1981/M6426) morphological form development in the gut of *Nyssomyia antunesi*: (**A**) elongated nectomonad; (**B**) short nectomonad; (**C**) metacyclic promastigote; (**D**) rounded metacyclic promastigote; (**E**) rounded paramastigote; (**F**) haptomonad. N, nucleus; K, kinetoplast (stained by Giemsa). Bar = 10 μm.

On day 8 pbm, six promastigote morphotypes of *L.* (*V.*) *lindenbergi* (Figure 4) and *L.* (*V*.) *lainsoni* (Figure 5) were observed in the gut of *Ny. antunesi*: elongated nectomonad, short nectomonad, metacyclic promastigote, rounded metacyclic promastigote, rounded paramastigote and haptomonad.

#### Survival Curves

**Figure 6:**
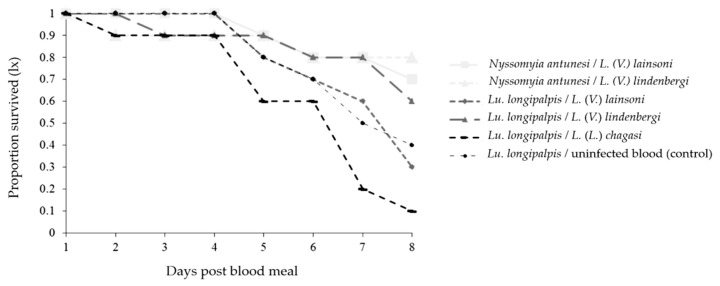
Survival curves of *Nyssomyia antunesi* and *Lutzomyia longipalpis* blood-fed on a *Leishmania* spp. suspension (or not) up to day 8 post-blood meal.

The survival of both phlebotomine species decreased up to day 8 pbm for all combinations (Figure 6). The survival of *Lu. longipalpis* was affected differently in some *Lu. longipalpis* combinations, and the survival was higher when the phlebotomine species were infected with *L.* (*V.*) *lindenbergi* than when they were infected with *L.* (*V.*) *lainsoni* (*p* < 0.0001) or *L.* (*L.*) *chagasi* (*p* < 0.0001). Moreover, the survival was higher when the phlebotomine species were infected with *L. (V.) lainsoni* compared with *L.* (*L.*) *chagasi* (*p* < 0.0001); and when they were infected with uninfected blood compared with those infected with *L.* (*V.*) *lindenbergi* (*p* = 0.0246), or *L.* (*L.*) *chagasi* (*p* < 0.0001) (Table 2).

## 4. Discussion

Several parasite–vector combinations were studied under laboratory conditions to evaluate interaction patterns, including the ability of vectors to support the late-stage development of parasites, suggesting the well-recognized classification of restrictive and permissive vectors. In the former category, phlebotomines present a remarkable specificity for a single (or some closely related) *Leishmania* species; while in the latter, phlebotomines allow for the development of a broad range of apart-related *Leishmania* species [35,36]. In this sense, the present study assessed *Ny. antunesi* and *Lu. longipalpis* in the development of medically important parasites in the Amazon biome, *L.* (*V.*) *lainsoni* and *L.* (*V*.) *lindenbergi,* until day 8 pbm, when late-stage promastigote forms were supposed to colonize the foregut, thus providing advanced inferences on their susceptibility.

After exhaustive attempts, the unsuccessful colonization of *Ny. antunesi* led the researchers to challenge wild-caught specimens. Although the unknown life status of these specimens and an apparently low number of assessments may compromise experimental reproducibility, it is believed that the field background brought about by nature adds pivotal elements for genuine parasite–vector interactions. *Nyssomyia antunesi* has been recognized as a suspected vector of *L.* (*V.*) *lindenbergi* based on some eco-epidemiological evidence [19,20,21], although no natural infection has been ascribed to this parasite. The present findings demonstrate that *Ny. antunesi* can develop at least a small population of late-stage promastigotes of both *L.* (*V.*) *lainsoni* and *L.* (*V.*) *lindenbergi* (a taxonomically distinct species) with no difference in parasitosis on day 8 pbm, suggesting that this species could be further investigated as a possible permissive vector, although it has never been found to be naturally infected by *L.* (*V*.) *lainsoni* in wild-caught specimens that have been examined. Interestingly, the present findings support early microscopic and current molecular-based evidence, which suggests that *Ny. antunesi* can harbor *Trypanosoma* sp. [37,38], *Porcisia* sp. [39], and a wide range of *Leishmania* spp. [40,41,42,43,44,45,46,47,48,49]. Most of these detections do not provide evidence of late-stage promastigote forms, which are insufficient to characterize *Ny. antunesi* as a true vector. Other supporting information for the present results is related to O-glycosylated proteins with N-acetylgalactosamine (GalNAc) epitopes, which are likely reported exclusively for permissive species [35], as has been suggested to be present in the midgut epithelial cells of *Ny. antunesi* [50].

On day 8 pbm, *L.* (*V.*) *lainsoni* and *L.* (*V.*) *lindenbergi* were observed up to the cardia of *Ny. antunesi,* probably because these *Leishmania* species need more time to advance to the stomodeal valve under laboratory conditions, as has been suggested for the binomial *Phlebotomus arabicus*–*L.* (*L.*) *infantum* [15]. On several occasions, it was noted that *Ny. antunesi* only partially fed under experimental conditions (Sánchez-Uzcátegui, personal observation), which could possibly be important information from an epidemiological point of view. In this sense, multiple bloodmeals during a single gonadotrophic cycle have been reported for *Lu. longipalpis* [51], which has a potential impact on survival and *Leishmania* transmission, as suggested by Killick-Kendrick [52], for *Ph. papatasi* and *L. major.* Moreover, this characteristic would improve vector competence since the development of a successful infection in wild phlebotomines is a gradual process that depends on the parasite’s action, which is amplified and enhanced by the ingestion of multiple blood meals [53]. Thus, all these facts add weight to the hypothesis that *Ny. antunesi* is an important vector from a medical point of view, but other criteria still need to be evaluated.

When evaluating the experimental infection of *Lu. longipalpis* with *L.* (*V.*) *lainsoni* and *L.* (*V.*) *lindenbergi,* it has been demonstrated that both parasite species can develop up to day 8 pbm with no difference in parasitosis between these combinations. Preliminary experimental infections have already been performed and the descriptions of these experiments with the two *Leishmania* species have focused on determining the developmental pattern for taxonomic purpose [19,38], thus not extending to the observation of late-stage promastigote forms. *Lutzomyia longipalpis* is well known as the major natural vector of *L.* (*L.*) *chagasi* [54], which is laboratory-supported as a permissive vector and competent for experimentally transmitting *L.* (*V*.) *braziliensis* [55], *L.* (*L.*) *chagasi* [56], *L.* (*L.*) *mexicana* [57], *L.* (*L.*) *major* [58,59] and *L.* (*L.*) *amazonensis* [60].

On day 8 pbm, parasitosis in *Lu. longipalpis* was higher in *L.* (*L.*) *chagasi* than in *L.* (*V.*) *lainsoni* and *L.* (*V.*) *lindenbergi*, reinforcing the status of the ancient and well-established *L.* (*L.*) *chagasi–Lu. longipalpis* natural binomial [54,61], herein regarded as the control experiment. Naturally, this combination [6,56,62,63,64] is overexploited due to its medical importance, as well as the manageable laboratory adaptation of *Lu. longipalpis* and consequent successful establishment of colonies [65], with effective rates of artificial blood feeding [22,24,66]. In addition, the results from developmental studies of *L.* (*L.*) *chagasi* in other phlebotomine species were also verified [67,68,69].

As expected, the predominant gut development reported for the studied *Leishmania* species (i.e., peripylarian for *L.* (*V.*) *lainsoni* and *L.* (*V.*) *lindenbergi*, and suprapylarian for *L.* (*L.*) *chagasi*) is in agreement with the taxonomic positions of these species originally described by Lainson and Shaw [34]. Few specimens with hindgut development were recorded for all combinations, which was exclusively attributed to heavy parasite loads throughout the phlebotomine gut. On that gut site, only adhered promastigotes were considered, avoiding artifactual observation due to back-wash [70]. In contrast, in the midgut, free-living promastigotes were considered. Killick-Kendrick [71] has shown striking features separating metacyclic forms from others in the phlebotomine gut, including a lack of attachment to the epithelium, high motility, a small body size and the presence of a long free flagellum.

The survival curves significantly decreased for all combinations, but were differentially affected in *Lu. longipalpis*–parasite combinations and uninfected blood. The reduced longevity of experimentally *Leishmania*-infected phlebotomines was documented [30,72,73], without evidence of a strain-specific impact [30]. Moreover, the results reported herein support the classical hypothesis that successful transmission in nature also depends on equilibrate parasitosis, which is sufficient for the inoculum but within the limits of vector tolerance, preserving its longevity [74].

## 5. Conclusions

In summary, wild-caught *Ny. antunesi* and laboratory-bred *Lu. longipalpis* have been suggested to be experimentally susceptible to *L.* (*V*.) *lainsoni* and *L.* (*V*.) *lindenbergi*, which results in the development of at least a small population of late-stage parasites up to day 8 pbm in the cardia or stomodeal valve. The putative permissiveness of *Ny. antunesi* has not been discarded, however, still requiring further assessment. The unsuccessful establishment of life cycles with these parasite–vector combinations in nature may result from non-negligible ecological field-driven elements. Indeed, the putative susceptibility of phlebotomines suggested herein is worthy of important epidemiological consequences because it enables a successful adaptation of *Leishmania*. A successful colonization of *Ny. antunesi* could provide a considerable number of specimens that allow for an in vivo and in vitro assessment of *Leishmania*–phlebotomine interactions, and thus definitively determine its permissiveness and vector competence status.

## Figures and Tables

**Table 1:** Summary statistics for the comparison of parasitosis on day 8 post-blood meal in different parasite–vector combinations. Significant differences are highlighted in bold.

Vector	Parasite	*n*	Statistics
*Ny. antunesi*	*L. (V.) lainsoni*	12	*G test* = 2.2148, *df* = 3, *p* = 0.5290
	*L. (V.) lindenbergi*	11	
*Lu. longipalpis*	*L. (V.) lainsoni*	42	*G test* = 1.7129, *df* = 3, *p* = 0.6341
	*L. (V.) lindenbergi*	58	
*Lu. longipalpis*	*L. (V.) lindenbergi*	58	*G test* = 8.636, *df* = 3, ***p* = 0.0345**
	*L. (L.) chagasi*	58	
*Lu. longipalpis*	*L. (V.) lainsoni*	42	*G test* = 8.0092, *df* = 3, ***p* = 0.0458**
	*L. (L.) chagasi*	58	

**Table 2:** Log-rank test significance on the comparison of survival curves obtained for the different parasite–vector combinations up to day 8 post-blood meal. Significant differences are highlighted in bold.

Vector	Parasite	*n*	Log-Rank Test Significance
*Ny. antunesi*	*L. (V.) lainsoni*	16	*p* = 0.6014
	*L. (V.) lindenbergi*	10	
*Lu. longipalpis*	*L. (V.) lainsoni*	159	***p* < 0.0001**
	*L. (V.) lindenbergi*	142	
*Lu. longipalpis*	*L. (V.) lindenbergi*	142	***p* < 0.0001**
	*L. (L.) chagasi*	94	
*Lu. longipalpis*	*L. (V.) lainsoni*	159	***p* < 0.0001**
	*L. (L.) chagasi*	94	
*Lu. longipalpis*	*Uninfected blood*	78	***p* = 0.0246**
	*L. (V.) lindenbergi*	142	
*Lu. longipalpis*	*Uninfected blood*	78	*p* = 0.0954
	*L. (V.) lainsoni*	159	
*Lu. longipalpis*	*Uninfected blood*	78	***p* < 0.0001**
	*L. (L.) chagasi*	94	

## Data Availability

All data supporting the conclusions are included within the article. The datasets used and/or analyzed during the current study are available from the corresponding author upon reasonable request.

## References

[1] Ready P.D. (2013). Biology of phlebotomine sand flies as vectors of disease agents. Annu. Rev. Entomol..

[2] Organização Pan-Americana da Saúde (OPAS)/Organização Mundial da Saúde (OMS) Leishmanioses. https://www.who.int/es/news-room/fact-sheets/detail/leishmaniasis.

[3] Becvar T., Vojtkova B., Siriyasatien P., Votypka J., Modry D., Jahn P., Modri D., Jahn P.P., Bates P., Carpenter S. (2021). Experimental transmission of *Leishmania* (*Mundinia*) parasites by biting midges (Diptera: Ceratopogonidae). PLoS Pathogens..

[4] Sunantaraporn S., Thepparat A., Phumee A., Sor-Suwan S., Boonserm R., Bellis G., Siriyasatien P. (2021). *Culicoides Latreille* (Diptera: Ceratopogonidae) as potential vectors for *Leishmania martiniquensis* and *Trypanosoma* sp. in northern Thailand. PLoS Negl. Trop. Dis..

[5] Ticha L., Kykalova B., Sadlova J., Gramiccia M., Gradoni L., Volf P. (2021). Development of various *Leishmania* (*Sauroleishmania*) *tarentolae* strains in three Phlebotomus species. Microorganisms.

[6] Freitas V.C., Parreiras K.P., Duarte A.P.M., Secundino N.F., Pimenta P.F. (2012). Development of *Leishmania* (*Leishmania*) *infantum chagasi* in its natural sandfly vector *Lutzomyia longipalpis*. Am. J. Trop. Med. Hyg..

[7] Borovsky D., Schlein Y. (1987). Trypsin and chymotrypsin-like enzymes of the sandfly *Phlebotomus papatasi* infected with *Leishmania* and their possible role in vector competence. Med. Vet. Entomol..

[8] Pimenta P.F.P., Modi G.B., Pereira S.T., Shahabuddin M., Sacks D.L. (1997). A novel role for the peritrophic matrix in protecting *Leishmania* from the hydrolytic activities of the sand fly midgut. Parasitology.

[9] Pimenta P.F., Turco S.J., McConville M.J., Lawyer P.G., Perkins P.V., Sacks D.L. (1992). Stage-specific adhesion of *Leishmania* promastigotes to the sandfly midgut. Science.

[10] Kamhawi S., Ramalho-Ortigao M., Pham V.M., Kumar S., Lawyer P.G., Turco S.J., Barillas-Mury C., Sacks D.L., Valenzuela J.G. (2004). A role for insect galectins in parasite survival. Cell.

[11] Lawyer P.G., Ngumbi P.M., Anjili C.O., Odongo S.O., Mebrahtu Y.B., Githure J.I., Koech D.K., Roberts C.R. (1990). Development of *Leishmania major* in *Phlebotomus duboscqi* and *Sergentomyia schwetzi* (Diptera: Psychodidae). Am. J. Trop. Med. Hyg..

[12] Sacks D.L. (1989). Metacyclogenesis in *Leishmania* promastigotes. Exp. Parasitol..

[13] Rogers M.E., Chance M.L., Bates P.A. (2002). The role of promastigote secretory gel in the origin and transmission of the infective stage of *Leishmania mexicana* by the sandfly *Lutzomyia longipalpis*. Parasitology.

[14] Nieves E., Pimenta P.F. (2000). Development of *Leishmania (Viannia) braziliensis* and *Leishmania* (*Leishmania*) *amazonensis* in the sand fly *Lutzomyia migonei* (Diptera: Psychodidae). J. Med. Entomol..

[15] Myskova J., Votypka J., Volf P. (2008). *Leishmania* in sand flies: Comparison of quantitative polymerase chain reaction with other techniques to determine the intensity of infection. J. Med. Entomol..

[16] Gonçalves L.P., Santos T.V.D., Campos M.B., Lima L.V.D.R., Ishikawa E.A.Y., Silveira F.T., Ramos P.K.S. (2020). Further insights into the eco-epidemiology of American cutaneous leishmaniasis in the Belem metropolitan region, Pará State, Brazil. Rev. Soc. Bras. Med. Trop..

[17] Ward R.D., Lainson R., Shaw J.J. (1977). Experimental transmissions of *Leishmania mexicana amazonesnis* Lainson & Shaw, between hamsters by the bite of *Lutzomyia flaviscutellata* (Mangabeira). Trans. R. Soc. Trop. Med. Hyg..

[18] Vasconcelos dos Santos T., Silveira F.T. (2020). Increasing putative vector importance of *Trichophoromyia* phlebotomines (Diptera: Psychodidae). Mem. Inst. Oswaldo Cruz..

[19] Silveira F.T., Ishikawa E.A.Y., De Souza A.A.A., Lainson R. (2002). An outbreak of cutaneous leishmaniasis among soldiers in Belém, Pará State, Brazil, caused by *Leishmania (Viannia) lindenbergi* n. sp.—A new leishmanial parasite of man in the Amazon region. Parasite.

[20] Pimentel A.C., Sánchez Uzcátegui Y.D.V., De Lima A.C.S., Silveira F.T., Vasconcelos dos Santos T., Ishikawa E.A.Y. (2022). Blood Feeding Sources of *Nyssomyia antunesi* (Diptera: Psychodidae): A Suspected Vector of *Leishmania* (Kinetoplastida: Trypanosomatidae) in the Brazilian Amazon. J. Med. Entomol..

[21] Sánchez Uzcátegui Y.D., Vasconcelos Dos Santos T., Silveira F.T., Ramos P.K., Dos Santos E.J.M., Póvoa M.M. (2020). Phlebotomines (Diptera: Psychodidae) from a urban park of Belém, Pará State, northern Brazil and potential implications in the transmission of American cutaneous leishmaniasis. J. Med. Entomol..

[22] Ready P.D. (1978). The feeding habits of laboratory-bred *Lutzomyia longipalpis* (Diptera: Psychodidae). J. Med. Entomol..

[23] Cabrera O.L., Munstermann L.E., Cárdenas R., Gutiérrez R., Ferro C. (2002). Definición de las condiciones de temperatura y almacenamiento adecuadas en la detección de ADN de *Leishmania* por PCR en flebotominos. Biomedica.

[24] Sánchez Uzcátegui Y.D.V., Dos Santos E.J.M., Matos E.R., Silveira F.T., Vasconcelos dos Santos T., Póvoa M.M. (2022). Artificial blood-feeding of phlebotomines (Diptera: Psychodidae: Phlebotominae): Is it time to repurpose biological membranes in light of ethical concerns?. Parasites Vectors.

[25] Vaselek S., Volf P. (2019). Experimental infection of *Phlebotomus perniciosus* and *Phlebotomus tobbi* with different *Leishmania tropica* strains. Int. J. Parasitol..

[26] Rowton E.D., Dorsey K.M., Armstrong K.L. (2008). Comparison of in vitro (chicken-skin membrane) versus in vivo (live hamster) blood-feeding methods for maintenance of colonized *Phlebotomus papatasi* (Diptera: Psychodidae). J. Med. Entomol..

[27] Munstermann L.E., Marquardt W.H. (2005). Care, Maintenance, and Experimental Infection of Phlebotomine Sand Flies in Biology of Disease Vectors.

[28] Lawyer P.G., Meneses C., Rowland T., Rowton E.D. (2016). Sand fly rearing. Care and Maintenance of Phlebotomine Sand Flies.

[29] Volf P., Volfova V. (2011). Establishment and maintenance of sand fly colonies. J. Vector Ecol..

[30] Agrela I.F., Feliciangeli M.D. (2015). Effect of *Leishmania* spp. infection on the survival, life expectancy, fecundity and fertility of *Lutzomyia longipalpis* sl. and *Lutzomyia pseudolongipalpis*. Mem. Inst. Oswaldo Cruz..

[31] Rabinovich J.E., Eva V. (1978). Mortalidad y tablas de vida. Ecología de Poblaciones Animales.

[32] Ayres M., Junior A.M. (2000). BioEstat 20: Aplicações Estatísticas nas Áreas das Ciências Biológicas e Médicas p. xii–259.

[33] De Souza A.A.A., Da Rocha Barata I., Silva M.D.G.S., Lima J.A.N., Jennings Y.L.L., Ishikawa E.A.Y., Prévot G., Ginouves M., Silveira F.T., Shaw J. (2017). Natural *Leishmania (Viannia)* infections of phlebotomines (Diptera: Psychodidae) indicate classical and alternative transmission cycles of American cutaneous leishmaniasis in the Guiana Shield, Brazil. Parasite.

[34] Lainson R., Shaw J.J., Peters W., Killick-Kendrick R. (1987). Evolution, classification and geographical distribution. The Leishmaniases in Biology and Medicine.

[35] Volf P., Peckova J. (2007). Sand flies and *Leishmania*: Specific versus permissive vectors. Trends Parasitol..

[36] Cecílio P., Cordeiro-da-Silva A., Oliveira F. (2022). Sand flies: Basic information on the vectors of leishmaniasis and their interactions with *Leishmania* parasites. Commun. Biol..

[37] Lainson R., Shaw J.J., Lumsden W.H.R., Evans D.A. (1979). The role of animals in the epidemiology of South American Leishmaniasis. Biology of the Kinetoplastida.

[38] Silveira F.T., Souza A.A., Lainson R., Shaw J.J., Braga R.R., Ishikawa E.A. (1991). Cutaneous leishmaniasis in the Amazon region: Natural infection of the sandfly *Lutzomyia ubiquitalis* (Psychodidae: Phlebotominae) by *Leishmania* (*Viannia*) *lainsoni* in Pará State, Brazil. Mem. Inst. Oswaldo Cruz..

[39] Thies S.F., De Morais Bronzoni R.V., Michalsky É.M., Dos Santos E.S., Da Silva D.J.F., Dias E.S., Damazo A.S. (2018). Aspects on the ecology of phlebotomine sand flies and natural infection by *Leishmania hertigi* in the Southeastern Amazon Basin of Brazil. Acta Trop..

[40] Ryan L., Silveira F.T., Lainson R., Shaw J.J. (1984). Leishmanial infections in *Lutzomyia longipalpis* and *Lu. antunesi* (Diptera: Psychodidae) on the island of Marajó, Pará State, Brazil. Trans R Soc. Trop. Med. Hyg..

[41] Vásquez-Trujillo A., Santamaría-Herreño E., González-Reina A.E., Buitrago-Álvarez L.S., Góngora-Orjuela A., Cabrera-Quintero O.L. (2008). *Lutzomyia antunesi* as suspected vector of cutaneous leishmaniasis in the Orinoquian region of Colombia. Rev. Salud Publica.

[42] Vásquez-Trujillo A., Reina A.E.G., Orjuela A.G., Suárez E.P., Palomares J.E., Alvarez L.S.B. (2013). Seasonal variation and natural infection of *Lutzomyia antunesi* (Diptera: Psychodidae: Phlebotominae), an endemic species in the Orinoquia region of Colombia. Mem. Inst. Oswaldo Cruz..

[43] Thies S.F., Ribeiro A.L.M., Michalsky E.M., Miyazaki R.D., Fortes-Dias C.L., Fontes C.J.F., Dias E.S. (2013). Phlebotomine sandfly fauna and natural *Leishmania* infection rates in a rural area of Cerrado (tropical savannah) in Nova Mutum, State of Mato Grosso in Brazil. Rev. Soc. Bras. Med. Trop..

[44] Chagas A.P., Soares D.C., De Sousa G.C.R., Viana R.B., Rebelo J.M.M., Garcez L.M. (2016). Aspectos ecológicos da fauna de flebotomíneos em focos de leishmaniose na Amazônia Oriental, Estado do Pará, Brasil. Rev. Pan-Amaz. Saude.

[45] Ogawa G.M., Pereira Júnior A.M., Resadore F., Ferreira R.D.G.M., Medeiros J.F., Camargo L.M.A. (2016). Sandfly fauna (Diptera: Psychodidae) from caves in the state of Rondônia, Brazil. Rev. Bras. Parasitol. Vet..

[46] De Oliveira Leão P., Júnior A.M.P., De Paulo P.F.M., Carvalho L.P.C., Souza A.B.N., Da Silva M.S., Castro T.S., De Souza M.T.F., De Souza M.M.R., Melim G.E.F. (2020). Vertical stratification of sand fly diversity in relation to natural infections of *Leishmania* sp. and blood-meal sources in Jamari National Forest, Rondônia State, Brazil. Parasit. Vectors.

[47] Araujo-Pereira T.D., Pita-Pereira D.D., Baia-Gomes S.M., Boité M., Silva F., Pinto I.D.S., De Sousa R.L.T., Fuzari A., De Souza C., Brazil R. (2020). An overview of the sandfly fauna (Diptera: Psychodidae) followed by the detection of *Leishmania* DNA and blood meal identification in the state of Acre, Amazonian Brazil. Mem. Inst. Oswaldo Cruz..

[48] Costa G.S.G., Júnior A.M.P., Castro T.S., De Paulo P.F.M., Ferreira G.E.M., Medeiros J.F. (2021). Sand fly fauna and molecular detection of *Leishmania* species and blood meal sources in different rural environments in western Amazon. Acta Trop..

[49] Carneiro A.C.G., De Souza E.A., Barroso E.P., De Ávila M.M., Melchior L.A.K., Rocha R.D.C., Shimabukuro P.H.F., Galati E.A.B., Brilhante A.F. (2022). Phlebotomine Fauna (Diptera: Psychodidae) and Infection by *Leishmania* spp. in Forest Fragments of a University Campus, Western Amazon. J. Med. Entomol..

[50] De Oliveira D.M.S., Da Silva B.J.M., De Sena C.B.C., Lima J.A.N., Dos Santos T.V., Silveira F.T., Silva E.O. (2016). Comparative analysis of carbohydrate residues in the midgut of phlebotomines (Diptera: Psychodidae) from colony and field populations from Amazon, Brazil. Exp. Parasitol..

[51] Elnaiem D.E.A., Morton I., Brazil R., Ward R.D. (1992). Field and laboratory evidence for multiple blood feeding by *Lutzomyia longipalpis* (Diptera: Psychodidae). Med. Vet. Entomol..

[52] Killick-Kendrick R., Lumsden W.H.R., Evans A. (1979). The biology of *Leishmania* in phlebotomine sandflies. Biology of the Kinetoplastida.

[53] Serafim T.D., Coutinho-Abreu I.V., Oliveira F., Meneses C., Kamhawi S., Valenzuela J.G. (2018). Sequential blood meals promote *Leishmania* replication and reverse metacyclogenesis augmenting vector infectivity. Nat. Microbiol..

[54] Lainson R., Rangel E.F. (2005). *Lutzomyia longipalpis* and the eco-epidemiology of American visceral leishmaniasis, with particular reference to Brazil: A review. Mem. Inst. Oswaldo Cruz..

[55] Coelho M.D.V., Falcão A.R. (1962). Experimental transmission of *L. brasiliensis.* I. Transmission through inoculation of *P. longipalpis* triturates. Rev. Inst. Med. Trop..

[56] Lainson R., Ward R., Shaw J. (1977). Experimental transmission of *Leishmania chagasi*, causative agent of neotropical visceral leishmaniasis, by the sandfly *Lutzomyia longipalpis*. Nature.

[57] Da Costa S.G., Moraes C.D.S., Bates P., Dillon R., Genta F.A. (2019). Development of *Leishmania mexicana* in *Lutzomyia longipalpis* in the absence of sugar feeding. Mem. Inst. Oswaldo Cruz..

[58] Myskova J., Svobodova M., Beverley S.M., Volf P. (2007). A lipophosphoglycan-independent development of *Leishmania* in permissive sand flies. Microbes Infect..

[59] Cecílio P., Pires A.C.A., Valenzuela J.G., Pimenta P.F., Cordeiro-da-Silva A., Secundino N.F., Oliveira F. (2020). Exploring *Lutzomyia longipalpis* sand fly vector competence for *Leishmania major* parasites. J. Infect. Dis..

[60] Silva R.C.R.D., Cruz L.N.P.D., Coutinho J.M.D.S., Fonseca-Alves C.E., Rebêlo J.M.M., Pereira S.R.F. (2021). Experimental transmission of *Leishmania (Leishmania) amazonensis* to immunosuppressed mice through the bite of *Lutzomyia longipalpis* (Diptera: Psychodidae) results in cutaneous leishmaniasis. Rev. Inst. Med. Trop..

[61] Silveira F.T., Corbett C.E.P. (2010). *Leishmania chagasi* Cunha & Chagas, 1937: Indigenous or introduced? A brief review. Rev. Pan-Amaz. Saúde.

[62] Soares R.P., Macedo M.E., Ropert C., Gontijo N.F., Almeida I.C., Gazzinelli R.T., Pimenta P.F.P., Turco S.J. (2002). *Leishmania chagasi*: Lipophosphoglycan characterization and binding to the midgut of the sand fly vector *Lutzomyia longipalpis*. Mol. Biochem. Parasitol..

[63] Ranasinghe S., Rogers M.E., Hamilton J.G., Bates P.A., Maingon R.D. (2008). A real-time PCR assay to estimate *Leishmania chagasi* load in its natural sand fly vector *Lutzomyia longipalpis*. Trans R Soc. Trop. Med. Hyg..

[64] Secundino N.F., De Freitas V.C., Monteiro C.C., Pires A.C.A., David B.A., Pimenta P.F. (2012). The transmission of *Leishmania infantum chagasi* by the bite of the *Lutzomyia longipalpis* to two different vertebrates. Parasit. Vectors.

[65] Lawyer P., Killick-Kendrick M., Rowland T., Rowton E., Volf P. (2017). Laboratory colonization and mass rearing of phlebotomine sand flies (Diptera, Psychodidae). Parasite.

[66] Killick-Kendrick R., Leaney A.J., Ready P.D. (1977). The establishment, maintenance and productivity of a laboratory colony of *Lutzomyia longipalpis* (Diptera: Psychodidae). J. Med. Entomol..

[67] Saraiva L., Carvalho G.M., Gontijo C.M., Quaresma P.F., Lima A.C., Falcão A.L., Filho J.D.A. (2009). Natural infection of *Lutzomyia neivai* and *Lutzomyia sallesi* (Diptera: Psychodidae) by *Leishmania infantum chagasi* in Brazil. J. Med. Entomol..

[68] Guimarães V.C.F.V., Pruzinova K., Sadlova J., Volfova V., Myskova J., Filho S.P.B., Volf P. (2016). *Lutzomyia migonei* is a permissive vector competent for *Leishmania infantum*. Parasit. Vectors.

[69] Galvis-Ovallos F., Ueta A.E., Marques G.D.O., Sarmento A.M.C., Araujo G., Sandoval C., Tomokane T.Y., Da Matta V.L.R., Laurenti M.D., Galati E.A.B. (2021). Detection of *Pintomyia fischeri* (Diptera: Psychodidae) with *Leishmania infantum* (Trypanosomatida: Trypanosomatidae) promastigotes in a focus of visceral leishmaniasis in Brazil. J. Med. Entomol..

[70] Lainson R., Ward R.D., Shaw J.J. (1977). *Leishmania* in phlebotomine sandflies: VI. Importance of hindgut development in distinguishing between parasites of the *Leishmania mexicana* and *L. braziliensis* complexes. Proc. R Soc. Lond B Biol. Sci..

[71] Killick-Kendrick R. (1990). The life-cycle of *Leishmania* in the sandfly with special reference to the form infective to the vertebrate host. Ann. Parasitol. Hum. Comparée.

[72] El Sawaf B.M., El Sattar S.A., Shehata M.G., Lane R.P., Morsy T.A. (1994). Reduced longevity and fecundity in *Leishmania*-infected sand flies. Am. J. Trop. Med. Hyg..

[73] Rogers M.E., Bates P.A. (2007). *Leishmania* manipulation of sand fly feeding behavior results in enhanced transmission. PLoS Pathog..

[74] Hamilton J.G.C., Hurd H., Lewis E.E., Campbell J.F., Sukhdeo M.V. (2002). Parasite manipulation of vector behaviour. The Behavioural Ecology of Parasites.

